# Female perspectives on male involvement in a human-papillomavirus-based cervical cancer-screening program in western Kenya

**DOI:** 10.1186/s12905-019-0804-4

**Published:** 2019-08-08

**Authors:** Konyin Adewumi, Sandra Y. Oketch, Yujung Choi, Megan J. Huchko

**Affiliations:** 1 Department of Obstetrics and Gynecology, Duke University, 2301 Erwin Road, Durham, NC 27710 UK; 2 Center for Microbiology Research, Kenya Medical Research Institute, P.O. Box 54840 00200, Off Mbagathi Road, Nairobi, Kenya; 3 Duke Global Health Institute, 310 Trent Dr, Durham, NC 27710 UK

**Keywords:** Male involvement, Cervical cancer, HPV, Kenya

## Abstract

**Background:**

To be effective, population-based cervical cancer prevention programs must be tailored to meet the needs of the target population. One important factor in cervical cancer screening may include male involvement. To iteratively improve a screening program employing self-collected vaginal swabs for human-papillomavirus (HPV) testing in western Kenya, we examined the role of male partners and community leaders in decision-making and accessing screening services.

**Methods:**

We carried out 604 semi-structured, in-depth interviews (IDIs) with women and community health volunteers who took part in a multiphase trial of implementation strategies for HPV-based cervical cancer screening. IDIs were coded and themes related to decision-making, screening and treatment barriers, and influence of male partners and community leaders were identified and analyzed.

**Results:**

Women experienced both support and opposition from their male partners. Partner support took the form of financial support for transportation and emotional support and encouragement, while opposition ranged from anticipated negative reactions to lack of permission, isolation, and abandonment. Though most women described their own partners as supportive, many felt that other male partners would not be supportive. Most participants believed that increased HPV and cervical cancer knowledge would increase partner support. Women reported a general acceptance of involvement of community leaders in education and screening campaigns, in a setting where such leaders may hold influence over men in the community.

**Conclusion:**

There was a clear interest in involving male partners in the cervical cancer prevention process, specifically in increasing knowledge and awareness. Future research should explore the feasibility and effectiveness of engaging male partners in cervical cancer screening and prevention programs.

**Electronic supplementary material:**

The online version of this article (10.1186/s12905-019-0804-4) contains supplementary material, which is available to authorized users.

## Background

Cervical cancer is a major public health issue that disproportionately affects women living in low-and middle-income-countries (LMICs). Despite advances in prevention through vaccination and screening programs, cervical cancer continues to be the fourth most common cancer worldwide [1]. Efforts to increase vaccination access will be crucial to long-term cervical cancer control in LMICs. However, vaccination roll-out in LMICs has been limited, and the effects on cervical cancer incidence will lag up to two years behind implementation. Effective population-based screening coupled with linkage to early treatment of precancerous lesions therefore remain critical to preventing the development of cervical cancer in the region.

While many LMICs have adopted the World Health Organization’s recommendations for cervical cancer screening, there remain large gaps in uptake of screening and linkage to follow-up for women who screen positive [2]. As programs are implemented in settings with limited health care infrastructure, there may be logistical barriers such as limited access to health facilities, costs, or a shortage of trained professionals. Compounding these challenges to access, population risk perception and screening knowledge is low in many areas [3, 4]. Studies have shown that educational interventions and community based cervical cancer prevention campaigns can increase knowledge and uptake of screening and treatment services [4, 5]. In such settings, low uptake, despite educational interventions and community-based screening, suggests that additional psychological, social or interpersonal barriers may prevent women in LMICs from accessing care.

Male partners may have a broad influence across the logistical, educational and psychosocial factors that influence women’s decisions around and uptake of screening. Male involvement may include positive facilitators, such as emotional support, encouragement, and financial support. However, it may also have negative impacts, such as stigmatization, isolation, or outright prohibition of access to care. In previous decades, sexual and reproductive health (SRH) initiatives have focused almost exclusively on women, often offering services in places where only women frequent. While strategies that incorporate gender issues are important, the direct involvement of male partners in SRH conversations has become increasingly recognized as a key factor in acceptance and uptake of care [6]. Studies have shown that both women and men are interested in shared decision-making in reproductive matters, linking partner influence to women’s adherence to HIV prevention methods, contraceptive use, and an increase in financial and interpersonal support for antenatal care and treatment during obstetric emergencies [7–13]. Some researchers remain critical of the efficacy of male involvement and its relationship to female empowerment, citing the absence of a standard definition of “male involvement”, and the need for a clearer understanding of the positive and negative outcomes of involving male partners in SRH programs [8, 14, 15]. Such criticisms may be addressed through the development and use of an analytical framework that focuses on the impact of male involvement. Preliminary evidence for the effectiveness of male involvement in cervical cancer screening in east Africa comes from a Ugandan study where male partners were shown a letter after their partners screened for HPV. Results showed that when male partners received the letter, women’s likelihood of returning for treatment after a colposcopy increased [16, 17]. While promising, there is limited research exploring the extent of male partner influence and the potential benefits and drawbacks of male involvement in cervical cancer prevention, including questions of acceptability and desirability. Given the gap in research and the known benefits of male involvement in other SRH issues, we explored the perceptions of women who participated in a cervical cancer prevention study in order to (1) identify key themes to inform the development of a cervical cancer prevention specific framework for male involvement and (2) identify key characteristics that may inform a more standard definition of “male involvement” with respect to HPV/cervical cancer prevention, including where (if at all) men should be included in the process.

## Methods

We used data from semi-structured, in-depth interviews with women and community health volunteers (CHVs) who took part in a multiphase trial of implementation strategies for human papillomavirus (HPV)-based cervical cancer screening in Western Kenya to examine perceptions regarding male involvement in cervical cancer prevention [18, 19]. In the parent study, women were offered HPV testing via self-collected vaginal swabs, in either community-health campaigns or government-supported clinics, with referral for treatment to the county hospital in the town of Migori, located approximately 92 km from the farthest screening site. Women did not receive compensation for transport or monetary incentives for their participation in the study. Participants and all community health volunteers (CHVs) were asked to participate in semi-structured in-depth interviews (IDIs) at various time points in the study.

We performed a qualitative analysis on 604 IDIs from four sets of IDIs with women and one set of IDIs with CHVs in the study communities that took part in the parent study. The IDIs were composed of four categories; (1) women that participated in the outreach and education campaign (n = 120), (2) women that were screened (n = 111), (3) women that received treatment (n = 283), (4) women that were considered non-adherent or lost to follow-up (LTFU) because they did not seek treatment at the county hospital within 60 days of receiving their results(n = 72). CHVs were interviewed at the completion of the study (n = 18). We purposively sampled women from the 12 study communities that participated in the education and screening campaigns. Study staff identified eligible women and contacted them by phone or home visit until ten women per community were interviewed. We sought to interview all treated women and a random sample of 81 women who were lost to follow-up but successfully interviewed 72.

IDIs with women consisted of open-ended questions about what they understood about HPV and treatment for HPV, their attitudes and experience with screening or treatment, barriers and facilitators to screening and treatment services, stigma and privacy, and the role of male partners in facilitating cervical cancer prevention. CHV interviews consisted of questions that explored their own perceived facilitators and challenges as well as those of the communities they served. The interview guides were designed as part of the parent study and included questions about challenges to community sites and health facilities, the overall logistics of the study, and their experiences with women who participated in the study. The interview guides are provided in Additional file 1, Additional file 2, Additional file 3, Additional file 4, and Additional file 5.

IDI guides were developed in English, then translated and conducted in either Dholuo or Kiswahili by researchers fluent in these languages. IDIs, conducted by trained study staff, were audio recorded onto tablets, and then transcribed and translated to English, and reviewed by the study coordinator for accuracy and adherence to the transcript. IDIs were coded by a team of four researchers using NVIVO 11™ software (QSR International, London, United Kingdom). The team developed a codebook using the interview guide and transcripts from five IDIs from each set. This codebook was tested with five different IDIs, followed by another round of discussion and revisions until the team agreed on a final codebook. For final coding, all interviews were coded by two separate researchers and then compared for agreement across coders.

We defined male involvement as the inclusion of male partners (either husbands or long-term boyfriends), male family members, or community members in any aspect of a screen-and-treat model for cervical cancer prevention. For preliminary analysis, coding reports were reviewed collaboratively to identify important themes relating to male involvement such as experiences and perceptions of partner support and opposition, including emotional and tangible (such as finances) support, stigma, lack of support, and partner opposition. Analysis emphasized male *partner* involvement because it was the most cited form of male involvement in the interviews. Interviews were further analyzed to determine if, how, and to what extent a male partner influences a woman’s screening and treatment process. We used findings from the data to develop a framework that describes ways in which male involvement could potentially influence women’s decision-making processes in an HPV-based cervical cancer-screening program, highlighting ways in which male involvement was both positive and negative. We then used this framework to structure and organize our results. Responses were sorted into categories that identified: (1) both perceived and experienced barriers; (2) experiences with male partners as facilitators to care; and (3) proposed facilitators to positive male involvement.

### Ethical Approval

This study was approved by the ethical review boards of the Kenya Medical Research Institute, Duke University and the University of California, San Francisco. All participants gave written informed consent before participation in the study.

## Results

A total of 604 IDIs, completed during the parent study, were used for this analysis. The majority of women interviewed reported being in a long-term relationship (detailed participant characteristics can be found in the parent study [18, 19]). Most participants cited male involvement as male partner involvement, which they believed was important in cervical cancer prevention. Whereas most women reported that the partner’s permission was not necessary and the decision to seek screening or treatment was their own, CHVs indicated that despite receiving information about HPV and cervical cancer, many women remained reluctant to seek screening without first obtaining their partner’s permission: “*… they still did not want to screen. Sometimes one tells you that she has to speak to her husband first (CHV IDI).”* Major themes that emerged throughout the interviews were (1) perceived male partner distrust and stigmatization, (2) importance and mechanisms of partner support, and (3) implications of limited partner health education. Themes were grouped into categories related to perceived or experienced barriers, male partners as facilitators, and suggestions on how to facilitate male involvement using the male involvement framework (explained above).

### Male Partners as Perceived or Experienced Barriers to Care

Although most women considered their own partners as “supportive”, many were of the view that male partner opposition was a major barrier to HPV prevention among other women in their communities. Women and CHVs cited HPV-related stigma, the cost of transportation to treatment facilities, and indifference towards women’s lives as their primary perceptions and experiences of male partners as barriers. Women who were in the “lost-to-follow-up” cohort tended to describe their own partners as a barrier to seeking treatment more often than women in the treatment arm. Importantly, women who were lost-to-follow-up and did not explicitly identify their male partners as a “barrier”, still expressed the view that (1) their partners did not take actions supportive of their attempts to seek treatment, such as encouragement, childcare or transport money or (2) they did not tell their male partners about their HPV positive test result.

### Partner Distrust/Opposition and Stigma

A lack of trust emerged as a key challenge among women who perceived or experienced male partner opposition. Distrust manifested in various ways throughout the analysis; including women’s distrust of their partners, male’s distrust and stigmatization of women, and a general distrust of the test/results. Women who cited distrust as a barrier to care reported that they were (1) unable to disclose their screening attendance or test results to their partners or (2) received negative responses when/ if they did choose to disclose. Women who reported that they felt that they could not disclose tended to report attending screening or treatment campaigns in secret. For some women this led to an inability to seek services, either from being prohibited from or not supported to reach the treatment site or a general inability to leave their homes.

*“I don’t know about travelling. You know we have husbands, I am a second wife and sometimes my husband never allows me to go anywhere. So, when [a community site] comes near us, the way it did, I can go and he will not find out that I went or took part (Screening IDI).”*

“*If a man hears that it is sexually transmitted, most of them jump to the conclusion that this woman went somewhere and got it because he thinks that he does not have it (CHV IDI).”*

Some women reported experiencing a lack of trust from their partners after disclosing their HPV results. Partner distrust also included accusations or stigmatization women received from their partner after disclosing their HPV results. Negative responses included accusations of multiple sexual partners, often relating to HPV-related stigma.

*“When I got the message, I gave it to my husband together with the consent form that I was given. He then read this and told me that only those who have multiple sex partners can get HPV which made me worry, I then consoled myself and decided to come for treatment (Treatment IDI).”*

CHVs also voiced concerns about the potential for HPV-related stigma to have a negative impact on interpersonal relationships for women who sought screening or tested HPV positive. Stigmatizing attitudes toward HPV included an association with promiscuity, infidelity, and HIV. One CHV stated that she had encountered several women who complained that their husbands viewed them as “*promiscuous after having been found to be HPV positive (CHV IDI).”*

Such accounts of stigma were often cited in relation to stigma surrounding HIV.

*“There are those who support their wives to get treatment to save [their] life but there are those who will feel that they will be stigmatized just like for the HIV positive cases (Treatment IDI).”*

As highlighted in the quote below, partner distrust of the HPV screening and treatment process or of study participation surfaced as a theme throughout the interviews. One perceived reason for such partner opposition, mentioned by women, was the absence of visible HPV symptoms among some women who tested positive for HPV.

*“Most of the time men are good with what they can see for instance, ‘so-and-so’ is sick and bedridden. But when one can still walk like I do and has not felt any problem, if I tell him that such a thing is happening, give me money I go for treatment, [ … ] he may think that I am lying to him. So, it is not easy for men. They can even feel that I want to go somewhere and have come up with a lie. People can only agree that one is sick only when they are bedridden (LTFU IDI).”*

### Financial Control

Male partners were frequently seen as potentiating the infrastructure and logistical barriers, through their control of finances and decisions about whether to pay for transportation to the treatment site. Male partners functioned as active barriers to treatment access when they were (1) unwilling to provide funds for transportation or (2) if women felt unable to disclose their need for transportation money. Male partners could also have a more passive negative role if they were seen as the source of money for transport, but did not have the ability to pay for it.

*“It is better to keep silent because some men lack understanding and when they get to hear that you have an illness, you can even be thrown away from the house. Not all men [are supportive], because one knows that their wife needs treatment and they offer no support even transport means (Treatment IDI).”*

### Unwillingness to Adhere to Post Treatment Recommendations

Almost all women in the study identified their partners as unlikely to adhere to post-treatment recommendations for abstinence. Women were worried that partners would not believe the instructions from health professionals, “*I have been told not to have sex with my partner for one month, will my partner believe me (Treatment IDI)?”* Many women asked interviewers for help informing their partners about post-procedure abstinence, either in person, over the phone or with written documentation. Some participants asked study staff for documented evidence of these instructions or to call their partner and disclose the information on their behalf.

*“I had to tell the nurse to document it because my husband doesn’t like using protection … If he isn’t understanding you will have to have disagreements because he would insist on having his way, but if you explain to him and he understands that for you to be well again there are changes he will have to make, you wouldn’t have any form of disagreement. I can’t allow anybody to abuse my rights (Treatment IDI).”*

In addition to abstinence, women were also concerned about the unwillingness of male partners to use condoms for three months:

“*My question is about men who will not agree to use protection*, *because that was the instruction we were given during screening and after treatment. What if he refuses to use protection? How can you help to prevent HPV re-infection in this situation (Treatment IDI)?*”

#### Male Partner Support or Facilitation

As mentioned previously, almost all women and CHVs interviewed in this study believed that it was important to involve male partners in cervical cancer prevention. Male partner support was a central theme throughout the IDIs. Responses regarding “support” were organized into two categories depending on whether a woman (1) reported experiences of partner support or facilitation or (2) suggested ways in which male partners can be beneficial to prevention. This analysis found that reports of “male support” often involved permission from male partners to get screened or treated, financial support for transport to services, and encouragement and emotional support.

*“It is important to reach [men]... Knowledge is power... When men know about it, then it is going to be even easier for them to allow [women] to go for treatment because they shall have also known the impact [of cervical cancer] (Outreach and Education IDI).”*

### Experiences with Male Partners as Facilitators

Most women gave examples of emotional and logistical partner support as facilitators to both screening and treatment. While many stated that the decision to screen was their own, contrary to how CHVs described it, they also reported that they received encouragement or support from their partners. Language around emotional partner support for participation in prevention services varied, with women using terms that indicated permission (“*I decided on my own and also asked my husband if I could go”),* agreement (“*he agreed that I should go for testing*”) and encouragement (“*he told me to go, you know one might feel that you don’t want to go yet you have some other things you are doing before you go for treatment*”).One woman explained how her partner provided emotional and financial support throughout her time in the parent study:

*“My husband supported me. When it [the cervical cancer prevention campaign] was first announced, I told him and he urged me to go for screening because I didn’t know my status and then later when I was being screened, I gave the ones who were screening me his phone number so I was notified through his phone. So, after he got the message, he tried very hard to look for money that would enable me to come for treatment (Treatment IDI).”*

In addition to emotional support, women stated that their partners also provided financial support, particularly for transportation to the treatment facility, allowing women to overcome barriers related to the cost of transportation.

*“There was no money and I wanted to go for treatment and so my husband looked for money in order for me to go for treatment (Treatment IDI).”*

Participants attributed their partners’ support to both the *“knowledge”* that their partners had about HPV and the availability of free treatment. Women also used phrases such as “*cancer is a deadly disease”* and *“my life is important to him”,* as potential reasons why their husbands were supportive.

### Proposed Facilitators to Increased Male Involvement

Most women, irrespective of personal experience or perceptions, believed that male partners could play an important role in increasing access to HPV screening/treatment services as well as adherence to post-treatment advice. All participants suggested ways of increasing male partners’ involvement as a means of addressing barriers to care. The education of male partners emerged as the major theme throughout the interviews because most women and CHVs believed that men were unlikely to seek this information on their own. Responses included when to educate men, where, what topics to include, the importance of educating male partners, and ways to implement partner education.

CHVs expressed a perceived need to deliver accurate male partner education, as early as possible in the prevention process. As one CHV stated, *“I feel these men should be involved from the word ‘go’ [the start] (CHV IDI).”* In addition to early education, women also highlighted the need for continuous education, believing that partner education must be continuous to normalize HPV screening.

*“Then we should have frequent [education] and not just a once in a while thing like we did, going to a given community for two weeks then we are done. If we can incorporate this, we make it a routine such that when women go to a clinic, just like the way HIV is done, it is incorporated in the whole system such that when one goes to seek any kind of treatment at the hospital, they get tested for HIV (CHV IDI).”*

Much of the suggested educational content for male partners related to addressing the experiences of stigma and distrust that women mentioned in previous sections in this paper. In addition to de-stigmatization, women believed education should focus on raising partner awareness around the fact that treatment is available. Both women and CHVs believed that such knowledge may improve their access to services by increasing the chances of getting permission from their partners.

*“Maybe if one turns out to be HPV positive it will be good for the male partners at least to know that she went for the screening and she tested positive, maybe it would help them in terms of encouragement, or support financially to go for the treatment (CHV IDI).”*

*“Most men will not permit their wives to go for cervical cancer screening since they believe that cancer cannot be cured, so it is important that they learn more about cervical cancer more than women (Outreach and Education IDI).”*

Women believed that male involvement would raise awareness of men’s role in the transmission of HPV. Participants stated that men needed to “*know that they are the ones who give us this disease.”* Participants also reported that a better understanding of HPV transmission and cervical cancer prevention would help men understand the importance of treatment and compliance with post-treatment [abstinence] instructions meant to prevent complications, including reinfection.

*“When the results turn out positive, they will understand that it is a thing that involves both of them and so it will be easy for them to accompany them for the treatment, and this will help the woman in as far as the post-treatment measures she is supposed to take are concerned. (Treatment IDI).”*

When probed about possible ways to reach men, CHVs tended to cite “direct outreach.” Recommended content included general (and continuous) messaging about sexual health as well as information on relationship building.

*“When we go to the community, once we have the men around, we can even just carry the condoms, talk to them about prevention and distribute the same to them. With that, we would have taken a step towards preventing the spread of HPV (CHV IDI).”*

Although much of the conversation around male involvement revolved around partner involvement, participants reported that community leaders and village elders could be beneficial for spreading information about HPV and cervical cancer in the community. Community elder involvement, either male or female, was viewed as a possible facilitator to screening and treatment because they could “convey information” about screening and treatment options as well as “encourage men and women” to get treatment. Women believed that the influence of community leaders may be effective in increasing HPV/cervical cancer outreach and education because community elders have the greatest understanding of the communities, how they function, and how best to spread information. Women also believed that community leaders could receive and distribute outreach information most quickly and efficiently. Interestingly, very few women commented on a preferred gender of community leaders, perhaps because the perceived role of community leaders was strictly limited to increasing awareness or because it is accepted that community leaders are generally men. However, future research could explore the acceptability, feasibility, and effectiveness of using community leaders to provide cervical cancer information, especially to men.

*“Educating both women and men, not all the women share with their spouses what they hear about self-testing, the men will encourage their wives to go for screening, involving community leaders like the chiefs to spread the information, giving education and motivating the women because they love free things for example by giving lesos [traditional fabric] or T- shirts and this is memorable (CHV IDI).”*

### Framework Creation and Utilization

**Fig. 1:**
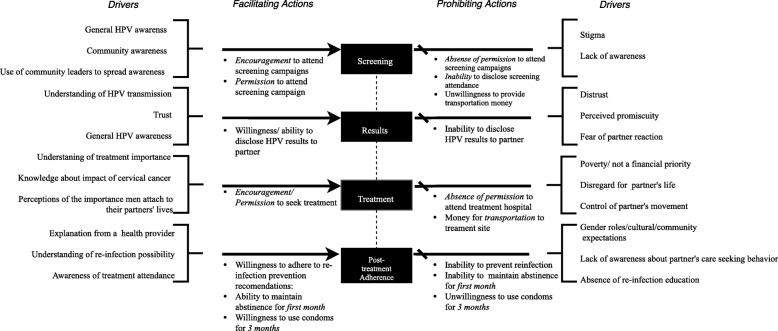
Framework for understanding male partner involvement in cervical cancer prevention programs

We used the perspectives of the participants to identify key themes, which informed the development of a cervical cancer prevention specific framework for male involvement. In particular, we sought to highlight where men were felt to impact the cervical cancer prevention process, including cervical cancer education, screening, results notification, and treatment. The various actions that served as barriers and facilitators mentioned with respect to each event highlighted in the cascade analyzed and explained in the text of this paper, are summarized in Fig. 1. “Drivers” were included to summarize the perceived causes or influencers of experienced or perceived barriers/facilitators. We included a category for “post treatment adherence” because of the large amount of data that emerged regarding barriers to post-treatment adherence.

## Discussion

To our knowledge, this is the first paper to explore female perspectives on “male involvement” related to cervical cancer in Western Kenya. Through qualitative interviews, we evaluated perspectives of women and CHVs who took part in a multiphase cervical cancer prevention study. We used their voices to develop a framework to describe the positive and negative ways in which male involvement impacts screening and treatment uptake. Through the relationships illustrated in the framework we were able to identify key drivers of facilitating actions and potential solutions to barriers as seen through women’s perspectives of male involvement. A key finding that informed the framework was the relationship between involvement and knowledge. Women who expressed a desire for increased male involvement in cervical cancer prevention, also perceived male partners to have limited knowledge of prevention. In contrast, women who reported having supportive partners also mentioned high levels of knowledge and awareness among their partners. Women identified ways in which male partners serve as facilitators and barriers to care as well as potential ways to ensure that male partner involvement could have a positive impact in the future, such as encouraging or permitting women to seek care.

Most women reported having supportive partners that provided emotional and tangible support; however, most participants and CHVs in general categorized male partners as actual or potential barriers to HPV screening and treatment access. Women who were considered lost-to-follow-up tended to cite their partners as barriers to treatment more often than others. Though all women reported a number of ways in which male partners could or did serve as barriers to care, distrust or lack of understanding were the most commonly identified manifestations of those barriers. Lack of support manifests itself through inability to provide financial or logistical support to travel for HPV screening or treatment.

Almost all women believed that educating male partners on cervical cancer prevention and treatment would lead to increased partner knowledge, enabling partners to better understand the treatment process. This is consistent with studies that have found that limited knowledge among male partners about cervical cancer may serve as a barrier to women accessing treatment [7, 20]. The majority of participants reported that targeting male partners during outreach and education activities in the early phase of prevention programs was the most feasible way to encourage an increase in male partner support. Educating male partners may equip men to better care for and interact with their partners; this included (1) permission/ encouragement to get screened or treated, (2) tangible support such as financial support for transport, (3) a reduction in partner distrust, and (4) an increase in adherence to post-treatment care. Women’s views regarding the relationship between men’s lack of knowledge of cervical cancer screening and their inability to provide support for cervical cancer screening or treatment indicate that defining male involvement in relation to cervical cancer will need standard interventions that emphasize the importance of partner education.

Our findings show that a lack of knowledge of cervical cancer prevention among male partners has potential negative influences on uptake of the services particularly in relation to women receiving permission/ financial assistance to undergo screening and treatment. This suggests a need for identifying strategies to effectively involve male partners in prevention programs. This may be particularly important as policy makers begin to place a greater emphasis on male involvement. For example, the World Health Organization’s ‘Comprehensive Cervical Cancer Control: A guide to essential practice’ addresses the need to involve male partners in cervical cancer prevention, as had been done in other aspects of reproductive health. Our findings are consistent with the WHO’s recognition of men as potential “gatekeepers” of access to services as well as its recommendation that increased knowledge among men also helps women make better health decisions [21].

Our findings regarding the potential role of community leaders in conducting male partner education are also consistent with the WHO’s recognition of such leaders playing an “essential role” in mobilizing communities and taking action to end men’s violence against women [21]. Evidence shows that the engagement of community leaders is a successful strategy for improving the response of communal justice mechanisms to violence against women and the prevention of trafficking of women and girls [21].

Importantly, the finding regarding male partners as a barrier to post-treatment care, especially prevention of complications or HPV re-infection through abstinence from sexual intercourse or condom use, highlights the need for culturally appropriate male partner education throughout the prevention cascade. Even the women who defined their partners as “supportive” expressed uncertainty or worry about their ability to convey to their partners the post-treatment recommendations of maintaining abstinence for one month and using a condom for three months after treatment. Potential partner opposition and distrust, especially in the context of reinfection prevention, point to the implications for future research, program, and policy decisions regarding where and how to involve men. As illustrated in the framework, education or targeted messaging of male partners would address some of the behaviors that stem from opposition and distrust.

It is important to note that while this study was able to sample a relatively under-studied population of women living in rural Kenya, particularly the women who had been lost to follow up and hard to find in other studies, there were some limitations [22]. Although IDIs were carried out by trained research assistants and women were assured that interviews were confidential, there remains the possibility of a social response bias, as well as reluctance to discuss negative personal experiences involving male partners. This potential weakness was accounted for in both the data collection and analysis process. The interviewers collecting the data were of the same cultural background as the participants; this likely contributed to the participants’ openness and willingness to discuss their experiences, including mentioning their partners. The analysis addressed this bias through the inclusion of (1) data where women reported perceived experiences rather than their own experience and (2) CHV perspectives. The use of CHV data provided objective insight into their personal experiences during their interactions with female clients and their partners throughout their involvement in the parent study. The finding regarding women’s emphasis on the importance of male involvement in cervical cancer prevention suggests a need for future studies to explore the perspectives of male partners, to (1) further understand ways in which male partners are and can be barriers and facilitators to prevention (2) understand how best to implement male partner education.

Finally, the main strength of the study was the development of a data-driven theoretical framework that highlights the importance and significance of understanding male partner involvement in cervical cancer prevention programs [23, 24]. In this article, we present an organized framework that systematically displays the possible relationship between the social and economic influence of male partners and women’s access to care. We believe that the data presented in this paper as well as the developed framework may begin to lay the foundations for (1) the assessment of potential barriers and facilitators within a cervical cancer prevention cascade and (2) the identification of a important factors that may define male involvement in cervical cancer prevention.

## Conclusions

The findings of this paper demonstrate an overwhelming desire for male partner education as well as a belief that increased male knowledge will lead to increased uptake of cervical cancer screening and prevention services. Most importantly, these findings highlight the need for greater conversations around male involvement that go beyond vaccination of boys in order to realize the global goal of eliminating cervical cancer by the year 2030.

## Additional files

Additional file 1: . Outreach and Education. (PDF 308 kb)

Additional file 2: Clinic Screening. (PDF 300 kb)

Additional file 3: Community Screening. (PDF 298 kb)

Additional file 4: Treatment. (PDF 299 kb)

Additional file 5: Female Clients. (PDF 381 kb)

## Data Availability

The datasets used and/or analyzed during the current study are available from the corresponding author upon reasonable request.

## Abbreviation

HPV: Human-papillomavirus (HPV)
IDIs: In-depth interviews (IDIs)
LMICs: Low-and middle-income-countries (LMICs)
LTFU: Lost to follow-up (LTFU)
SRH: Sexual and reproductive health (SRH)
