# Supplementary material for: Female perspectives on male involvement in a human-papillomavirus-based cervical cancer-screening program in western Kenya
Source: BMC Womens Health. 2019 Aug 8;19:107. doi: 10.1186/s12905-019-0804-4 (PMC6688365; doi:10.1186/s12905-019-0804-4)
Supplement: Supplementary file 1 — . Outreach and Education. (PDF 308 kb) [file 12905_2019_804_MOESM1_ESM.pdf]

Cluster Randomized Trial of Community-based Cervical Cancer Screening using HPV Testing

Female Client Open Interview:
Component 1 - HPV and Cervical Cancer Outreach and Education

Participant ID: ☐☐☐☐

Site ID: ☐☐

Community-based screening: ☐

Clinic-based screening: ☐

Interviewer Initials: ☐☐☐

Today's Date:

Day ☐☐ Month ☐☐ Year ☐☐

Time Interview Started: Hour ☐☐ Minutes ☐☐

Time Interview Ended: Hour ☐☐ Minutes ☐☐

	Yes	No
**Has the consent form been reviewed with the interviewee?**	☐	☐
**Has the consent form been signed and dated by the interviewee?**	☐	☐

INTERVIEWER SAY: *I want to thank you for taking the time to meet with me today. My name is ________ and I would like to talk to you about your experience participating in the HPV and Cervical Cancer Community Outreach Campaign and Education Module. First, I will ask you a series of questions that require short answers The second part of the interview will be more like a conversation where I will ask for your thoughts and opinions about completing the educational session. As we discussed in the consent process, I'd like to tape record your answers during the interview if that is okay with you.*

Section 1: Demographic Characteristics

No.	Questions	Answer Choices
101	How old are you?	Age in years: ☐☐
102	What is the higest level of education you've completed?	Standard 1 … ☐<br>Standard 2 … ☐<br>Standard 3 … ☐<br>Standard 4 … ☐<br>Standard 5 … ☐<br>Standard 6 … ☐<br>Standard 7 … ☐<br>Standard 8 … ☐<br>Form 1 ………….. ☐<br>Form 2 ………….. ☐<br>Form 3 ………….. ☐<br>Form 4 ………….. ☐<br>College …………. ☐<br>University ……… ☐<br>Beyond University ☐<br>None …………… ☐

103	What is your occupation?	Professional/technical/managerial ……. ☐<br>Domestic service ………………………... ☐<br>Agriculture and Fishing ………………… ☐<br>Clerical …………………………………… ☐<br>Sales and services ……………………... ☐<br>Skilled manual …………………………… ☐<br>Unskilled manual ………………………... ☐<br>None / Housewife .... ☐
104	How far is your home from the village center?	Distance in kilometres: ☐☐
105	How far is your home from the district hospital?	Distance in kilometres: ☐☐
106	What is your relationship status?	Single .... ☐<br>Married/Partnered ……… ☐<br>Separated .... ☐<br>Widowed/Divorced …….. ☐
107	How many children do you currently have?	Number of children: ☐☐
108	Are you pregnant?	Yes ………… ☐<br>No …………… ☐
109	If pregnant, what is your estimated due date?<br>*If not pregnant, skip to question #110.*	Day ☐☐ Month ☐☐ Year ☐☐
110	Are you currently using a method of family planning?	Yes ………… ☐<br>No …………… ☐
111	If you are using FP, what method(s) are you using? (Check all that apply).<br>*If not using FP, skip to question #112.*	Male condom ... ☐ Female condom ……… ☐<br>Injectable/Depo ☐ Sterilization …………… ☐<br>Birth control pills ☐ Abstinence …………… ☐<br>IUCD ………… ☐ Natural family planning ☐<br>Implant ……….. ☐
112	Have you previously been screened for cervical cancer?	Yes ………… ☐<br>No …………… ☐

113	During what year were you screened for cervical cancer?<br><br>*If not previously screened, skip to question #116*.	☐☐☐☐
114	What type of cervical cancer screening did you undergo?	VIA/VILI ………… ☐<br>Pap Smear ……… ☐<br>HPV ……………… ☐<br>Unknown .... ☐
115	What was the result of the cervical cancer screening test?	Positive .... ☐<br>Negative .... ☐<br>Unknown ……….. ☐
116	Have you previously been treated for cervical cancer or precancer?	Yes ………… ☐<br>No …………… ☐
117	Please describe the type of treatment you received.<br><br>*If not previously treated, skip to question #118.*	________________<br>________________<br>________________
118	What was the date of your last HIV test?	Day ☐☐ Month ☐☐ Year ☐☐<br><br>Never been tested for HIV …….. ☐
119	What was the result of your last HIV test?<br><br>*If never tested, skip to question #122.*	Positive .... ☐<br>Negative .... ☐
120	If HIV positive, are you in HIV care?	Yes ………… ☐<br>No …………… ☐
121	Where are you receiving HIV care?<br><br>*If not in HIV care, skip to question #122.*	________________<br>________________
122	Please describe any medical conditions that you have.	________________<br>________________<br>________________<br>________________

Section 2: Quantitative Questions about the Outreach and Education Component

No.	Question	Answer Choices
201	Was information about cervical cancer screening and HPV-based testing provided in your community?	Yes ………… ☐<br>No …………… ☐
202	If yes, where did you receive this information?<br><br>*If no, skip to next section.*	Market …………………………… ☐<br>Church ………………………….. ☐<br>Women's group meeting ……... ☐<br>Fliers/posters ………………….. ☐<br>Other …………………………… ☐<br>_______________________________
203	If yes, had you heard of cervical cancer screening previously, or was this the first time?	Heard about screening before ……….. ☐<br>First time hearing about screening …… ☐
204	Were you comfortable with the messages in the outreach and mobilization activities?	Yes ………… ☐<br>No …………… ☐
205	Were you comfortable with the people providing the outreach and mobilization activities?	Yes ………… ☐<br>No …………… ☐
206	How did you feel about the amount of time outreach and mobilization activities took?	The amount of time was appropriate …….. ☐<br>Too much time ……………………………… ☐<br>Not enough time …………………………… ☐
207	During the outreach activitiets, what topics were covered?<br><br>**INTERVIEWER**: *Do not read out the list. Check all that apply.*	Female anatomy …………………………… ☐<br>Cervical cancer …………………………….. ☐<br>HPV ………………………………………….. ☐<br>Cervical cancer screening ………………... ☐<br>Cervical cancer treatment ………………… ☐<br>How to perform HPV self-testing ………... ☐
208	At the end of the mobilization, did you receive information on how to access cervical cancer screening in your community?	Yes ………… ☐<br>No …………… ☐
209	Do you plan to seek cervical cancer screening?	Yes ………… ☐<br>No …………… ☐

210	If you don't plan to seek cervical cancer screening, why not?<br><br>*If yes, skip to next section.*	____<br><br>____

Section 3: Open-ended Questions

INTERVIEWER SAY: *As I have mentioned, I will now ask you some questions about your thoughts and opinions about the cervical cancer outreach campaign and educational module. By "outreach" I mean the advertisements and announcements about both the educational module and screening availability. By "educational module" I mean the brief session that covered specific cervical cancer topics. There are no right or wrong answers to these questions – your views will be very helpful to us as we develop cervical cancer prevention interventions in the future. I will be recording our interview as we go along.*

No.	Question
401	Where do you feel would be the most effective place for outreach campaigns?<br><br>*Probe: Where would outreach campaigns reach the most women? Why?*
402	What kinds of outreach activities would be most effective?<br><br>*Probes: What activities would reach the highest number of women?*<br><br>*What activities would women find easy to understand?*
403	What kinds of outreach activities would not be acceptable?<br><br>*Probes: What activities might make women uncomfortable?*<br><br>*What activities might offend women (or men)?*
404	Have you previously heard of cervical cancer programs in your own or nearby communities, and if so, can you describe some of the outreach activities?
405	Do you believe it is important for outreach campaigns to also reach men? Why or why not?
406	What do you feel is the level of knowledge about cervical cancer prevention among those in your community?<br><br>*Probe: Among women, men, health care providers?*
407	How can cervical cancer education for women be improved?
408	What role does cervical cancer education play in a woman's decision to get screened?
409	Who do you feel would be most appropriate to conduct community outreach and to teach the education module?

	Probe: Is it important for outreach and education workers to be a certain gender, or to have medical training?
410	What settings do you think are most appropriate for a detailed education module on cervical cancer?<br><br>*Probe: Is the education module appropriate for group settings like clinic bays/waiting areas?*
413	Do you have any other thoughts about the outreach campaigns and education module that we haven't covered?

INTERVIEWER: *Thank respondent for her time and assistance.*

Return to front page and enter ending time.

Interviewer notes or observations:
