# Supplementary material for: Female perspectives on male involvement in a human-papillomavirus-based cervical cancer-screening program in western Kenya
Source: BMC Womens Health. 2019 Aug 8;19:107. doi: 10.1186/s12905-019-0804-4 (PMC6688365; doi:10.1186/s12905-019-0804-4)
Supplement: Supplementary file 2 — Clinic Screening. (PDF 300 kb) [file 12905_2019_804_MOESM2_ESM.pdf]

Cluster Randomized Trial of Community-based Cervical Cancer Screening using HPV Testing

Female Client Open Interview: Component 2 – Clinic-based Screening

Participant ID: ☐☐☐☐

Site ID: ☐☐

Clinic screening site: ________________

Interviewer Initials: ☐☐☐

Today's Date:

Day	Month	Year
☐☐	☐☐	☐☐

Time Interview Started:

Hour	Minutes
☐☐	☐☐

Time Interview Ended:

Hour	Minutes
☐☐	☐☐

	Yes	No
**Has the consent form been reviewed with the interviewee?**	☐	☐
**Has the consent form been signed and dated by the interviewee?**	☐	☐

INTERVIEWER SAY: *I want to thank you for taking the time to meet with me today. My name is ________ and I would like to talk to you about your experience participating in the HPV testing program. First, I will ask you a series of questions that require short answers The second part of the interview will be more like a conversation where I will ask for your thoughts and opinions about completing the educational session. As we discussed in the consent process, I'd like to tape record your answers during the interview if that is okay with you.*

Section 1: Demographic Characteristics

No.	Questions	Answer Choices
101	How old are you?	Age in years: ☐☐
102	What is the higest level of education you've completed?	Standard 1 … ☐<br>Standard 2 … ☐<br>Standard 3 … ☐<br>Standard 4 … ☐<br>Standard 5 … ☐<br>Standard 6 … ☐<br>Standard 7 … ☐<br>Standard 8 … ☐<br>Form 1 ………….. ☐<br>Form 2 ………….. ☐<br>Form 3 ………….. ☐<br>Form 4 ………….. ☐<br>College ………… ☐<br>University ……… ☐<br>Beyond University ☐<br>None …………… ☐

103	What is your occupation?	Professional/technical/managerial ……. ☐<br>Domestic service ………………………... ☐<br>Agriculture and Fishing ………………… ☐<br>Clerical …………………………………… ☐<br>Sales and services …………………….... ☐<br>Skilled manual …………………………… ☐<br>Unskilled manual ………………………... ☐<br>None / Housewife .... ☐
104	How far is your home from the village center?	Distance in kilometres: ☐☐
105	How far is your home from the district hospital?	Distance in kilometres: ☐☐
106	What is your relationship status?	Single .... ☐<br>Married/Partnered ……… ☐<br>Separated .... ☐<br>Widowed/Divorced …….. ☐
107	How many children do you currently have?	Number of children: ☐☐
108	Are you pregnant?	Yes ………… ☐<br>No …………… ☐
109	If pregnant, what is your estimated due date?<br>*If not pregnant, skip to question #110.*	Day ☐☐ Month ☐☐ Year ☐☐

Section 2: Quantitative Questions about the Clinic-based Screening Component

No.	Question	Answer Choices
201	How did you travel to the clinic?	Walked …………. ☐<br>Public Means …………… ☐<br>Private Vehicle………….. ☐<br>Other…………………….. ☐
202	How far did you travel from your home to the clinic?	Distance in kilometres: ☐☐

203	Did you find the clinic location to be a satisfactory location for HPV testing?	Yes .... ☐<br>No .... ☐
204	Did you have adequate privacy at the clinic when you conducted the HPV testing?	Yes .... ☐<br>No .... ☐
205	Did you elect for HPV self-testing or for a provider to conduct the test?	Self-testing .... ☐<br>Provider conduct test .... ☐
206	If you asked for a clinician to conduct the test, why?<br>*If self-tested, skip to question #207.*	____________________<br>____________________
207	Were you able to complete the HPV self-testing?<br>*If provider conducted test, skip to question #214.*	Yes .... ☐<br>No .... ☐
208	If you were unable to complete the HPV self-testing, why? Check all that apply.<br>*If completed, skip to question #209.*	Menses .... ☐<br>Did not understand instructions .... ☐<br>Did not have adequate privacy .... ☐<br>Too uncomfortable/painful .... ☐<br>Not enough time .... ☐<br>Other ____________________
209	Were the self-testing instructions clear?	Yes .... ☐<br>No .... ☐
210	Was someone available to answer your questions about self-testing?	Yes .... ☐<br>No .... ☐
211	Was the self-testing uncomfortable?	Yes .... ☐<br>No .... ☐
212	Was the self-testing painful?	Yes .... ☐<br>No .... ☐
213	Would you test via self-collection again?	Yes .... ☐<br>No .... ☐
214	Would you recommend HPV testing to a friend?	Yes .... ☐<br>No .... ☐

215	At the end of the HPV testing, did you receive information on how to get your results?	Yes ………… ☐<br>No ………….. ☐
216	How do you prefer to receive your test result?	SMS ……………………….. ☐<br>Will return to my clinc …… ☐

Section 3: Open-ended Questions

INTERVIEWER SAY: *As I have mentioned, I will now ask you some questions about your thoughts and opinions about HPV testing and cervical cancer prevention.There are no right or wrong answers to these questions – your views will be very helpful to us as we develop cervical cancer prevention interventions in the future.*

No.	Question
301	What did you like most about the HPV self-testing?<br><br>*If client asked for a provider to conduct the test, skip to question #307.*
302	What did you not like about the HPV self-testing?
304	Do you feel that most women will be able to complete specimen collection in a single visit? Why or why not?
305	What can be done to facilitate the completion of HPV self-testing?<br><br>*Probe: Are there ways to make self-testing more acceptable or comfortable for women?*
306	Are there ways that the self-testing instructions can be improved (i.e., made easier to understand)?
307	Do you think HPV self-testing will be acceptable to women in your community? Why or why not?
308	If you had the option, would you prefer getting cervical cancer screening at community sites or at a local clinic? Why?
309	Do you feel like it is important for a clinician to be part of cervical cancer screening? Why or why not?
310	Who decided whether you would get screened for cervical cancer?
311	What are some factors that contributed to a your decision to get screened?<br><br>*Probes: Distance to clinic, comfort with screening test, male partner approval*
312	What could be done to encourage other women to get screened for cervical cancer?

313	Do you have any other thoughts about the HPV testing or cervical cancer prevention that we haven't covered?
**INTERVIEWER:** *Thank respondent for her time and assistance.*<br><br>*Return to front page and enter ending time.*	
**<u>Interviewer notes or observations:</u>**	
