# Supplementary material for: Female perspectives on male involvement in a human-papillomavirus-based cervical cancer-screening program in western Kenya
Source: BMC Womens Health. 2019 Aug 8;19:107. doi: 10.1186/s12905-019-0804-4 (PMC6688365; doi:10.1186/s12905-019-0804-4)
Supplement: Supplementary file 4 — Treatment. (PDF 299 kb) [file 12905_2019_804_MOESM4_ESM.pdf]

Cluster Randomized Trial of Community-based Cervical Cancer Screening using HPV Testing

Female Client Open Interview: Component 4 – Treatment

**Participant ID**:	☐☐☐☐	**Today's Date**: Day ☐☐ Month ☐☐ Year ☐☐	
**Site ID**:	☐☐		
**Community-based screening:**	☐	**Time Interview Started**: Hour ☐☐ Minutes ☐☐	**Time Interview Ended**: Hour ☐☐ Minutes ☐☐
**Clinic-based screening:**	☐		
**Interviewer Initials**:	☐☐☐		

	Yes	No
**Has the consent form been reviewed with the interviewee?**	☐	☐
**Has the consent form been signed and dated by the interviewee?**	☐	☐

INTERVIEWER SAY: *I want to thank you for taking the time to meet with me today. My name is ________ and I would like to talk to you about your experience receiving notification of your treatment for a positive HPV test result.First, I will ask you a series of questions that require short answers The second part of the interview will be more like a conversation where I will ask for your thoughts and opinions about completing the educational session. As we discussed in the consent process, I'd like to tape record your answers during the interview if that is okay with you.*

Section 1: Demographic Characteristics

No.	Questions	Answer Choices	
101	How old are you?	Age in years: ☐☐	
102	What is the higest level of education you've completed?	Standard 1 … ☐	Form 1 ………….. ☐
		Standard 2 … ☐	Form 2 ………….. ☐
		Standard 3 … ☐	Form 3 ………….. ☐
		Standard 4 … ☐	Form 4 ………….. ☐
		Standard 5 … ☐	College ………… ☐
		Standard 6 … ☐	University ………. ☐
		Standard 7 … ☐	Beyond University ☐
		Standard 8 … ☐	None ……………. ☐

103	What is your occupation?	Professional/technical/managerial ……. ☐<br>Domestic service ………………………... ☐<br>Agriculture and Fishing ………………… ☐<br>Clerical …………………………………… ☐<br>Sales and services ……………………... ☐<br>Skilled manual …………………………… ☐<br>Unskilled manual ………………………... ☐<br>None / Housewife .... ☐
104	How far is your home from the village center?	Distance in kilometres: ☐☐
105	How far is your home from the district hospital?	Distance in kilometres: ☐☐
106	What is your relationship status?	Single .... ☐<br>Married/Partnered ……… ☐<br>Separated .... ☐<br>Widowed/Divorced …….. ☐
107	How many children do you currently have?	Number of children: ☐☐

Section 2: Quantitative Questions about the Treatment of a Positive HPV Test Result

No.	Question	Answer Choices
201	How long after the notification of test results did you access treatment?	Time in days: ☐☐<br>Or<br>Time in weeks: ☐☐
202	How far did you have to travel to arrive at the treatment facility?	Distance in kilometres: ☐☐
203	What type of transportation did you use?	Walk ………….. ☐<br>Public means…………… ☐<br>Private vehicle …………. ☐<br>Other ________________________________
204	How much did the transportation cost?	________________________________

205	Did you receive transportation reimbursement for your trip(s) to the local treatment facility to access treatment?	Yes ………… ☐<br>No …………… ☐
206	Did you consider it easy or difficult to access treatment?	Easy ………… ☐<br>Difficult ……… ☐<br>☐ Neither………
207	What treatment did you receive?	Cryotherapy ……….. ☐<br>LEEP ……………….. ☐<br>Biopsy ……………... ☐<br>Unknown …………… ☐
208	Was the treatment explained clearly to you?	Yes ………… ☐<br>No …………… ☐
209	If you had cryotherapy or LEEP, were you able to complete your treatment in a single visit?	Yes ………… ☐<br>No …………… ☐
210	If you were unable to complete your treatment, why? Check all that apply.<br>*If completed treatment, skip to question #211.*	Menses ………………………………………… ☐<br>Inadequate supplies/equipment at site ……. ☐<br>No trained staff member …………………….. ☐<br>Changed my mind …………………………… ☐<br>Not enough time to wait ……………………… ☐<br>Complication during treatment…………….. ☐<br>Was referred to another hospital for cryotherapy or LEEP ……………. ☐<br>Other ______________________
211	How many visits did you need for treatment (cryotherapy or LEEP)?	Number of visits: ☐☐
212	Did you consider your treatment to be uncomfortable?	Yes ………… ☐<br>No …………… ☐
213	Did you consider your treatment to be painful?	Yes ………… ☐<br>No …………… ☐
214	Did you feel that you had adequate privacy during your treatment visit(s)?	Yes ………… ☐<br>No …………… ☐

215	Did anyone go with you to your treatment visit(s)? If yes, who?	Yes ………… ☐<br>No …………… ☐<br>If yes, who? ________________________________
216	Did you have to miss work to attend your treatment visit(s)?	Yes ………… ☐<br>No …………… ☐
216	If a friend had a positive HPV test, would you recommend treatment for them?	Yes ………… ☐<br>No …………… ☐

Section 3: Open-ended Questions

INTERVIEWER SAY: *As I have mentioned, I will now ask you some questions about your thoughts and opinions about the treatment for a positive HPV test result and cervical cancer prevention.There are no right or wrong answers to these questions – your views will be very helpful to us as we develop cervical cancer prevention interventions in the future.*

No.	Question
301	Can you describe what you have learned about treatment for a positive HPV test?
302	What comes to mind when you think about treatment for a positive HPV test?
303	Please describe your treatment experience.<br>*Probes: Was the treatment clearly explained to you? Did you understand the treatment?*<br>*Was the healthcare provider respectful?*<br>*Were you able to ask any questions, and if so, what questions did you ask?*<br>*Do you have any questions about treatment that you did not ask? What questions? Why didn't you ask?*<br>*Did you feel that you had adequate privacy during treatment?*<br>*Was treatment uncomfortable or painful?*<br>*Did you have any side effects after treatment? If so, please describe.*
304	Can you describe any factors that made accessing treatment difficult for you?<br>*Probes: Did you have to miss work?*<br>*Did you have house/childcare responsibilities?*<br>*Did you have enough money for transportation?*<br>*Was the hospital facility too far away?*<br>*Did you not want anyone else to know about the treatment?*

305	Are there ways that healthcare providers and treatment facilities can make treatment more comfortable for you? Please describe.
306	What would make it easier for women in your community to access treatment?<br><br>*Probes: Transporation vouchers, treatment "navigators", mobile treatment unites, SMS and phone reminders?*<br><br>*Involvement of community leaders, husbands/male partners?*
307	Do you feel that husbands/male partners are supportive of women accessing treatment? Why or why not?
308	If a friend got a positive HPV test, would you recommend that she get treated? Why or why not?
309	Do you have any other thoughts about the treatment of a positive HPV test results or cervical cancer prevention that we haven't covered?
**INTERVIEWER:** *Thank respondent for her time and assistance.*<br><br>*Return to front page and enter ending time.*	
**Interviewer notes or observations:**	
