# Supplementary material for: Female perspectives on male involvement in a human-papillomavirus-based cervical cancer-screening program in western Kenya
Source: BMC Womens Health. 2019 Aug 8;19:107. doi: 10.1186/s12905-019-0804-4 (PMC6688365; doi:10.1186/s12905-019-0804-4)
Supplement: Supplementary file 5 — Female Clients. (PDF 381 kb) [file 12905_2019_804_MOESM5_ESM.pdf]

# Cluster Randomized Trial of Community-based Cervical Cancer Screening using HPV Testing

## Female Client Open Interview:
## Component 5 – Treatment Loss to Follow Up

**Participant ID**: ☐☐☐☐

**Site ID**: ☐☐

**Community-based screening:** ☐

**Clinic-based screening:** ☐

**Interviewer Initials**: ☐☐☐

**Today's Date**:

| Day | Month | Year |
|---|---|---|
| ☐☐ | ☐☐ | ☐☐ |

| **Time Interview Started**: | | **Time Interview Ended**: | |
|---|---|---|---|
| Hour | Minutes | Hour | Minutes |
| ☐☐ | ☐☐ | ☐☐ | ☐☐ |

| | Yes | No |
|---|---|---|
| **Has the consent form been reviewed with the interviewee?** | ☐ | ☐ |
| **Has the consent form been signed and dated by the interviewee?** | ☐ | ☐ |

**INTERVIEWER SAY**: *I want to thank you for taking the time to meet with me today. My name is ________ and I would like to talk to you about your experience in cervical cancer screening and challenges in seeking treatment after the HPV test. First, I will ask you a series of questions that require short answers. The second part of the interview will be more like a conversation where I will ask for your thoughts and opinions about HPV testing and treatment. As we discussed in the consent process, I'd like to tape record your answers during the interview if that is okay with you.*

## Section 1: Demographic Characteristics

| No. | Questions | Answer Choices | |
|---|---|---|---|
| 101 | How old are you? | Age in years: ☐☐ | |
| 102 | What is the higest level of education you've completed? | Standard 1 … ☐ | Form 1 …………... ☐ |
| | | Standard 2 … ☐ | Form 2 …………... ☐ |
| | | Standard 3 … ☐ | Form 3 …………... ☐ |
| | | Standard 4 … ☐ | Form 4 …………... ☐ |
| | | Standard 5 … ☐ | College ………… ☐ |
| | | Standard 6 … ☐ | University ……… ☐ |
| | | Standard 7 … ☐ | Beyond University ☐ |

| | | |
|---|---|---|
| | | Standard 8 … ☐ None …………… ☐ |
| 103 | What is your occupation? | Professional/technical/managerial ……. ☐<br>Domestic service ………………………... ☐<br>Agriculture and Fishing ………………… ☐<br>Clerical …………………………………… ☐<br>Sales and services ……………………... ☐<br>Skilled manual …………………………… ☐<br>Unskilled manual ………………………... ☐<br>None / Housewife .............................. ☐ |
| 104 | What is your relationship status? | Single ........................... ☐<br>Married/Partnered ……… ☐<br>Separated ..................... ☐<br>Widowed/Divorced …….. ☐ |
| 105 | How many children do you currently have? | Number of children: ☐☐ |
| 106 | How far is your home from the village center? | Distance in kilometres: ☐☐ |
| 107 | How far is your home from the district hospital? | Distance in kilometres: ☐☐ |

**Section 2: Quantitative Questions about the Treatment of a Positive HPV Test Result**

| No. | Question | Answer Choices |
|---|---|---|
| 201 | Did you consider your screening to be physically uncomfortable or painful? | Yes ………… ☐<br>No …………… ☐<br>Don't know…… ☐ |
| 202 | Did you consider your screening to be embarrassing? | Yes ………… ☐<br>No …………… ☐<br>Don't know …. ☐ |
| 203 | Did you feel that you had adequate privacy during | Yes ………… ☐ |

| | | |
|---|---|---|
| | your screening visit? | No …………… ☐<br>Don't know….. ☐ |
| 204 | Did anyone go with you to your screening visit? | Yes ………… ☐<br>If yes, who? ______________________________<br>No …………… ☐ |
| 205 | Did you receive your HPV results?<br>If Yes ⟶ Go to Question 206<br>If No "I'm sorry about that. To make sure we have the correct number for you, what is your phone number?" Record ⟶ Skip to Q 213<br>If Don't know ⟶ Skip to Q 213 | Yes………………… ☐<br>No………………….. ☐<br>Phone Number: ________________________<br>Don't know………… ☐ |
| 206 | Did your HPV results make you feel worried? | Yes………………… ☐<br>No………………… ☐<br>Don't know………. ☐ |
| 207 | Did your HPV results make you feel afraid? | Yes………………… ☐<br>No……………….. ☐<br>Don't know………. ☐ |
| 208 | How were you notified of your results? | SMS ……………….. ☐<br>Phone Call………… ☐<br>Home Visit……….. ☐<br>Clinic Visit………… ☐ |
| 209 | What did you like about your notification option? | Nothing………………………………….. ☐<br>Liked confidentiality………………….. ☐<br>Convenience……………………………. ☐<br>Information about HPV……………….. ☐<br>Help with scheduling appointment ☐<br>Don't know……………………………… ☐<br>Other…………………………………….. ☐ |
| 210 | What did you least like about your notification option? | Nothing………………………………….. ☐<br>Worried about confidentiality………… ☐<br>Would prefer results face to face…….. ☐<br>Difficult scheduling appointment……… ☐<br>Don't know………………………………. ☐<br>Other …………………………………… ☐ |
| 211 | How long after screening were you notified of your HPV results? | < 1 week…………… ☐<br>1-2 weeks………… ☐ |

| | | |
|---|---|---|
| | | >2 weeks-1 month... ☐<br>> 1 month………… ☐<br>Don't know………… ☐ |
| 212 | Did you have all the information you needed to schedule an appointment for treatment? | Yes……………….. ☐<br>No………………… ☐<br>Don't know……… ☐ |
| 213 | What type of transportation would you use to get to Migori County Hospital? | Walk …………………….. ☐<br>Public means………….. ☐<br>Private vehicle ………… ☐<br>Other ……........................... ☐ |
| 214 | How much would the transportation cost you in total for a trip from your home to Migori and back? | KSH______________________ |
| 215 | Do you consider it easy or difficult to access treatment? | Easy ………… ☐<br>Difficult ……… ☐<br>Neither……… ☐ |
| 216 | Have you sought cervical cancer screening elsewhere since the HPV screening? | Yes ………… ☐<br>No ………….. ☐<br>Don't Know …. ☐ |
| 217 | Have you sought treatment for cervical cancer or precancer elsewhere since the HPV screening?<br>If Yes → Go to Q 218<br>If No → Skip to Q 219 | Yes ………… ☐<br>No ………….. ☐<br>Don't Know …. ☐ |
| 218 | What treatment was received? | Cryotherapy ☐<br>LEEP ☐<br>Hysterectomy ☐<br>Other................ ☐ |
| **INTERVIEWER SAY**: For the next few questions I will say a statement. Please tell me how much you agree or disagree with that statement. For example, if the first statement was 'The sky is blue' I would say I 'strongly agree' because I believe that is true. | | |
| 219 | I trust cervical cancer screenning to give accurate information about my health.<br>Do you…*(read answer choices)* | 1=Strongly agree,<br>2=Somewhat agree,<br>3=Somewhat disagree, or<br>4=Strongly disagree<br>8=Refused<br>9=Don't know |

| | | |
|---|---|---|
| 220 | If a cervical cancer screening test finds a problem, it can usually be treated. Do you…*(read answer choices)* | 1=Strongly agree<br>2=Somewhat agree<br>3=Somewhat disagree<br>4=Strongly disagree<br>8=Refused<br>9=Don't know |
| 221 | I would rather not know if I had cervical cancer. | 1=Strongly agree<br>2=Somewhat agree<br>3=Somewhat disagree<br>4=Strongly disagree<br>8=Refused<br>9=Don't know |
| 222 | If I had cervical cancer, people would think I slept around. | 1=Strongly agree<br>2=Somewhat agree<br>3=Somewhat disagree<br>4=Strongly disagree<br>8=Refused<br>9=Don't know |
| 223 | There is very little a person can do to reduce her risk of getting cervical cancer. | 1=Strongly agree<br>2=Somewhat agree<br>3=Somewhat disagree<br>4=Strongly disagree<br>8=Refused<br>9=Don't know |
| 224 | How worried are you about getting cervical cancer? Would you say you are…*(read answer choices)* | 1 = Not at all worried;<br>2 = A little worried;<br>3 = Moderately worried; or<br>4 = Very worried<br>8=Refused<br>9=Don't know |
| 225 | If you got cervical cancer, how much would it affect your life? Would it affect your life…*(read answer choices)* | 1 = Not at all;<br>2 = A little;<br>3 = A moderate amount; or<br>4 = A lot<br>8=Refused<br>9=Don't know |
| | **Section 3: Open-ended Questions** | |
| **INTERVIEWER SAY**: *As I have mentioned, I will now ask you some questions about your thoughts and opinions about HPV screening and treatment. There are no right or wrong answers to these questions – your views will be very helpful to us as we develop cervical cancer prevention interventions in the future.* | | |
| 301 | What factors contributed to your seeking HPV screening?<br><br>*Probes: Did you have a reproductive-health related illness?*<br>*Did your friend encourage you?*<br>*Do you perceive yourself at risk of cervical cancer?* | |
| 302 | Do you remember your HPV test result? What was it? Can you describe what your HPV test result means? (*If didn't receive result:* Can you describe what a positive HPV test result means?)<br><br>*Probes: Was the result clearly explained to you?*<br>*Did you understand the result?*<br>*How does this positive HPV test relate to your risk for cervical cancer?* | |

| | |
|---|---|
| 303 | Please describe your treatment options. (*If didn't receive result*: Can you describe the treatment options for a positive test result?)<br><br>*Probes: When can you go for treatment?*<br>*What does the treatment consist of?*<br>*Will the treatment cost money?*<br>*Do you know how to schedule an appointment?* |
| 304 | Do you plan to seek treatment? (*If didn't receive result:* If you had a positive test result, would you seek treatment? *If did go for treatment:* Why did you decide to seek treatment?)<br><br>*Probes: If not…Why?*<br>*What did you think about when deciding not to seek treatment?*<br>*If yes or already did…What caused you to delay seeking treatment?*<br>*What did you think about when deciding to seek treatment?* |
| 305 | How do you feel when you think about getting treatment?<br><br>*Probes: When you think about getting treatment, does it make you nervous, excited, overwhelmed, embarrassed, relieved, or any other emotions?* |
| 306 | (*If didn't receive result:* Can you describe any factors that would make accessing treatment difficult for yout)?Can you describe any factors that made accessing treatment difficult for you?<br><br>*Probes: Would you have to miss work, house, or childcare responsibilities?*<br>*Do you have enough money for transportation?*<br>*Is the hospital facility too far away?*<br>*Are you worried someone else would know you are going to get treatment?* |
| 307 | What would make it easier for women in your community to access treatment?<br><br>*Probes: Would the involvement of community leaders make it easier?*<br>*If yes, what would you like them to do?*<br>*What could your husband/male partner do to make treatment easier?*<br>*List any of the following she has not already mentioned. Would any of these be helpful for you? Transporation vouchers, treatment "navigators", mobile treatment units, SMS and phone reminders, friendlier health care providers.* |
| 308 | Do you feel that husbands/male partners are supportive of women accessing treatment? Why or why not?<br><br>*Probes: Do you know anyone whose partner was or would be angry that they had a + HPV test?*<br>*Do you know anyone whose partner would prevent them from getting treatment?*<br>*Did you tell your partner when you received your results?*<br>*What was your partner's reaction (remember, this is all confidential)?* |
| 309 | If a friend got a positive HPV test result, would you recommend that she get treated?<br><br>*Probe: Why or why not?* |
| 310 | Have you ever gone to Migori (or other hospitals) for care or treatment?<br><br>*Probes: If yes, what was your experience like?*<br>*Did you feel well-treated and respected?* |

| | |
|---|---|
| | *Was the cost of care or treatment high?*<br>*How did you get there? What was the journey like?* |
| 311 | Do you have any other thoughts about why someone who is HPV positive might not go for treatment and what would help her to go? Do you have any other thoughts about cervical cancer prevention that we haven't covered? |
| | **INTERVIEWER:** *Thank respondent for her time and assistance.*<br><br>*Return to front page and enter ending time.* |

**Interviewer notes or observations:**
